# Simple capacitor-switch model of excitatory and inhibitory neuron with all parts biologically explained allows input fire pattern dependent chaotic oscillations

**DOI:** 10.1038/s41598-020-63834-7

**Published:** 2020-04-30

**Authors:** Pavel Cejnar, Oldřich Vyšata, Jaromír Kukal, Martin Beránek, Martin Vališ, Aleš Procházka

**Affiliations:** 1 Department of Computing and Control Engineering, Faculty of Chemical Engineering, University of Chemistry and Technology in Prague, Prague, Czech Republic; 2 Department of Neurology, Faculty of Medicine in Hradec Králové, Charles University, Hradec Králové, Czech Republic; 3 Independent researcher, Uppsala, Sweden; 4 Czech Institute of Informatics, Robotics and Cybernetics, Czech Technical University in Prague, Prague, Czech Republic

**Keywords:** Biophysical models, Neural circuits

## Abstract

Due to known information processing capabilities of the brain, neurons are modeled at many different levels. Circuit theory is also often used to describe the function of neurons, especially in complex multi-compartment models, but when used for simple models, there is no subsequent biological justification of used parts. We propose a new single-compartment model of excitatory and inhibitory neuron, the capacitor-switch model of excitatory and inhibitory neuron, as an extension of the existing integrate-and-fire model, preserving the signal properties of more complex multi-compartment models. The correspondence to existing structures in the neuronal cell is then discussed for each part of the model. We demonstrate that a few such inter-connected model units are capable of acting as a chaotic oscillator dependent on fire patterns of the input signal providing a complex deterministic and specific response through the output signal. The well-known necessary conditions for constructing a chaotic oscillator are met for our presented model. The capacitor-switch model provides a biologically-plausible concept of chaotic oscillator based on neuronal cells.

## Introduction

Reasoning, resolution from facts are still the domains where the human brain outperforms current computers. This, but not only this, is the reason to model its functions, functions of neurons and information processing inside them. Neurons are modeled on many different levels of detail^1,2^ and for many different purposes. Models focusing on the information function of a neuron with varying levels of complexity^3–5^ laid the foundations for computer neural networks and are based on a simple mathematical calculus. Phenological models of the neuron^6–9^ have attempted to simulate more appropriately the output signal using more complicated mathematical formulas, however, mostly unrelated to existing biological structures and often only for the purpose of simulating nerve tissue alone^10^. Biological models^9,11,12^, on the other hand, describe in detail changes of individual quantities, ion currents, intracellular second messengers and ion pools in cells or in their compartments, but at the cost of high simulation complexity in available software tools like NEURON, GENESYS, or SNNAP^7,9,13–15^.

## The Function of Neuron

To understand the nature of information processing in neurons, we can focus on the differences in structure or function of neuronal cells relative to any other cells. Neuronal cells, localized in the brain, are protected from the rest of the body by the hematoencephalic barrier. This allows further functional specialization^16^. Although there are still some subcellular structures in neurons whose function is only assumed, like the Nissl substance, there are hardly any other undetected physical structures present that would not be identified at least by their physical location and shape to date. Morphologically, in a typical neuron, three major regions are recognized: (1) the soma, containing the nucleus and the major cytoplasmic organelles; (2) a variable number of dendrites (generally in submillimeter lengths each) or the dendritic tree, differing in size and shape, emanating from the soma to other neurons in gray matter and receiving signals from them through the synapses; and (3) the axon (up to lengths in meters in extremes^17^), which extends far from the cell body^16^. The important difference to other cells consists in the frequent changes in electropotential, the oscillation pattern, of the neurons. The potential acquired through synapses spreads through the dendritic tree to the soma and possibly undergoes some signal preprocessing operations. The received signal is thought to be integrated and if exceeding a threshold in the soma, an action potential is generated in the axon initial segment^18,19^ by opening Na^+^ ion channels and subsequently K^+^ ion channels. This change in potential, the spike, is then actively spread through the axon to other neurons. In the meantime, the resting electropotential is maintained using the ionic pumps by means of active transport of ions through the cell membrane.

### Electric Signals Recorded in Brain and Neuron, Excitatory and Inhibitory Neurons

Although no uniform classification scheme exists, many characteristic patterns among recorded oscillation patterns exist. “Regular spiking” generates one spike at a time for a longer period, such as in brain stem and spinal cord motor neurons. Sometimes, when a frequency seems to slow down in time, a “spike frequency adaptation” pattern is recognized, like in cortical and hippocampal pyramidal cells. Many neurons (thalamic relay neurons, inferior olivary neurons, some types of cortical and hippocampal pyramidal cells) are also able to exhibit “burst spiking”, with membrane potential increased through Ca^2+^ ion currents, accompanied by clusters of regular Na^+^- and K^+^ -dependent action potentials. However, among these often occurring patterns, many other exist, like γ-aminobutyric acid (GABA) releasing interneurons in the cerebral cortex, thalamus or hippocampus generating relatively short-duration (<l ms) action potentials and discharging at high frequencies (>300 Hz). Alternatively, neurons releasing neuromodulatory transmitters, such as acetylcholine, norepinephrine, serotonin, and histamine, often exhibit spontaneously generating action potentials at relatively slow frequencies (e.g., 1–10 Hz)^16^. *In vitro* recordings generally confirmed that each morphologically distinct class also differs in its electrophysiological features compared to other classes^20^.

We can divide neurons according to the changes in electropotential they spread and also according to the distance of their contacts to other neurons. In the brain we can recognize excitatory neurons making local contacts (e.g., spiny stellate cells of the cerebral cortex), excitatory neurons making distant contacts (e.g., pyramidal neurons in the cerebral cortex, spinal motor neurons), inhibitory neurons making local contacts (e.g., GABAergic interneurons in the cerebral and cerebellar cortex, in neocortex and hippocampus also classified by their morphology and function – basket cells, Chandellier cells, double bouquet cells, etc.), inhibitory neurons making distant contacts (e.g., medium spiny neurons of the basal ganglia or Purkinje cells of the cerebellar cortex) and, as a special category, neuromodulatory neurons (e.g., dopaminergic neurons of the substantia nigra) that influence the chemical transmission of the electropotential.

### Synapses Acting as a Mechanical Voltage-Gated Switch, Synaptic Delay, Passive Spread of Signal in Dendrites

Synapses consist of several components: (1) a presynaptic terminal localized often on axon, but alternatively also on dendrites (2) a cleft, and (3) a postsynaptic receptor site located on the dendrites, but alternatively also on axon, soma, or other types of cells^16^. Synapses allow transmission of the potential from the terminal to the receptor, and two types of synapses are recognized according to their transfer mechanism – electrical synapses and chemical synapses. Electrical synapses have smaller cleft (about 2–4 nm in comparison to 20–40 nm for chemical synapses^21^) and contain gap junction channels crossing the plasma membranes of both cells and allowing direct electrical coupling of both neurons^22^. Ions could travel both directions but sometimes, in rectifying synapses, voltage-gated ion channels allow traveling only in one direction^23^. Electrical synapses are often found connecting smaller local inhibitory neurons^24^, allowing them to synchronize^25^. Chemical synapses are found between both excitatory and inhibitory neurons and often occur as axo-dendritic synapses^26^. They can also connect axon with dendrites of the same cell. In the presynaptic area synapses contain neurotransmitters enclosed in small membrane-bound spheres – synaptic vesicles. According to the thickness of the presynaptic and postsynaptic area and to the shape of the vesicles, synapses also tend to be characterized as asymmetric (with rounded vesicles, typically excitatory synapses, found on dendritic spines) or symmetric (with flattened or elongated vesicles, typically inhibitory synapses, found on somata or dendritic shafts)^27^. The rise in potential on the presynaptic element activates voltage-dependent, calcium-selective ion channels^28^, influencing the membrane proteins and changing the shape of the membrane^29^ to release neurotransmitter from the vesicles to the cleft. On binding the neurotransmitter molecules from the cleft, the receptor site opens ligand-gated ion channels in the postsynaptic cell membrane, causing the ions to flow from or to the cell, which results in change of potential in the postsynaptic element. The change could also be modulated (amplified or inhibited) by chemical messengers produced inside the postsynaptic cell. At the end of the transmission, the neurotransmitter may be taken back to the presynaptic terminal by reuptake pumps, diffused away out of the cleft, or metabolized by the enzymes within the subsynaptic membrane^30^. The whole process allows unidirectional spread of the change in potential from presynaptic terminal to postsynaptic receptor site and, based upon neurotransmitter bound to the synapse, the detected significant increase in the potential of the presynaptic terminal (excitatory) or significant decrease (inhibitory) is propagated. The small volume of the cleft allows neurotransmitter concentration to be raised or lowered rapidly^23^, however, in comparison to electrical synapses, the whole process of transmission of the change of the potential is slower, although the difference is not as distinctive in mammalian neurons as in some cold-blooded animals^24^. The typical delay of 0.3–1 ms in the signal is generated due to releasing and binding of the neurotransmitter^11^. Both the electrical^31^ and chemical synapses^32,33^ undergo some long-term changes – exhihibing synaptic plasticity.

The electric features of dendrites, like the attenuation or delay computed from membrane and internal resistances, allow for spread of the signal in dendrites at short (approximately sub-millimeter) distances to other compartments of the dendritic trees^34–37^ at reasonably fast speeds, keeping in mind that these connections are not as persistent as axons, undergo changes during the neuronal cell life and, in the absence of significant insulation, their signal is susceptible to other dendro-dendritic signal preprocessing operations. A wide range of such operations is documented, like the logical AND/OR operations, coincidence detection, low-pass filter, attenuation, segregation, amplification or partial integration^38,39^.

### Ion Concentrations Inside and Outside The Cell, Ionic Pumps and The Resting Potential

**Figure 1:**
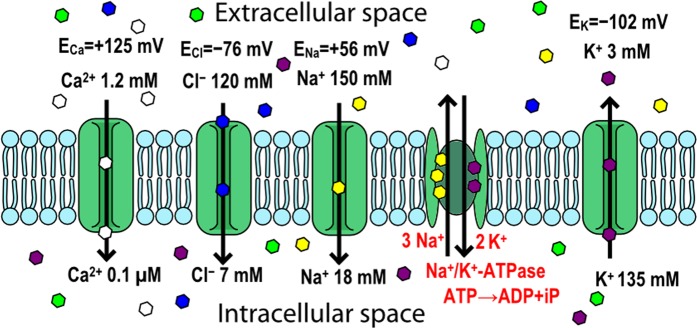
Different concentrations and equilibrium potentials for selected ions (Na^+^, K^+^, Cl^−^, and Ca^2+^) maintained inside and outside the cell, the example of ionic pump (Na^+^/K^+^-ATPase) for active transport against the concentration gradient and several passive ion channels for flow of selected ions down the concetration gradient.

The concentrations of important ions like Na^+^, K^+^, Cl^−^, or Ca^2+^ in neurons are precisely maintained by their active transport through the cell membrane using ionic pumps and then by several ionic buffering mechanisms. The intracellular and extracellular concentrations of these ions differ markedly: K^+^ is actively concentrated inside the cell, and Na^+^, Cl^−^, and Ca^2+^ are actively extruded out of the cell. However, while the plasma membrane is impermeant to larger and other ions, the osmolarity inside the cell is approximately equal to that outside the cell. Ions tend to passively move down their concentration gradients through specialized selective pores, ionic channels, if they are open in the plasma membrane. As ions travel through the membrane (e.g. K^+^ out of the cell), they slightly decrease the concentration gradient, whereas they significantly increase the electropotential gradient in the opposite direction. The equilibrium, where the concentration gradient of ions is balanced through their electropotential gradient, can be computed for given ion and its known concentrations on both sides of the membrane using the Nernst equation or, when incorporating other ions, using the Goldman–Hodgkin–Katz voltage equation. The equilibrium potentials for Na^+^, K^+^, Cl^−^, and Ca^2+^ ions, together with their intracellular and extracellular concentrations, are depicted in Fig. 1. In mammalian neurons, the equilibrium potential is approximately −102 mV for K^+^, 56 mV for Na^+^, −76 mV for Cl^−^, and 125 mV for Ca^2+^^16^. The change in ion concentration to reach the equilibrium potential is very small (in a spherical cell of 25 µm diameter, only about 4 µM for intracellular K^+^ (e.g., from 140 to 139.996 mM)). Intracellular recordings from different neurons in the mammalian central nervous system reveal different resting membrane potentials in a range of approximately −60 to −75 mV. And this could also change during wake/sleep period (e.g. approximately −70 mV during sleep and −55 mV during waking for cells receiving axonal input from the retina and projecting it to the visual cortex).

Among ionic pumps, the Na^+^/K^+^-ATPase is the most comprehensively understood^40^, extruding three Na^+^ ions for every two K^+^ ions transported into the cell at the cost of one ATP molecule. Other ionic pumps are used to selectively maintain the proper distribution of different ionic species important for cellular signaling and for secondary transport, being operated on the basis of the Na^+^ gradient across the cell membrane or of hydrolysis of ATP. For calcium, the concentration inside neurons is kept very low by both types of ionic pumps −Ca^2+^ is extruded from neurons through a Ca^2+^/Mg^2+^-ATPase and also through a Na^+^/Ca^2+^ exchanger driven by the Na^+^ gradient across the membrane. Low Cl^−^ concentration in neurons is actively maintained through a chloride-bicarbonate exchanger^16^. The number of the most common ionic pumps in human body cells, Na^+^ /K ^+^ -ATPase, differs by cell type but is generally between 8 · 10^5^ and 5 · 10^7^ ^41,42^.

The human brain consumes about 20% of the body’s oxygen supply at rest and utilizes approximately 25% of body glucose, even though it represents only about 2% of the body weight^16,43^. The most of the energy in neuronal cells is consumed for maintaining the gradient in concentration of selected ions and thus maintaining the difference in potential^44^.

### Ionic Generation of Action Potential, Axon Hillock, Axon Initial Segment

As recorded on the squid giant axon by Hodgkin and Huxley and explained using a quantitative mathematical model^12,45–47^, the small deviation from resting potential (of approximately −60 mV inside versus outside on squid giant axon) exceeding the activation threshold (approximately − 45 to −55 mV) leads first to the depolarization of membrane through opening of Na^+^ ion channels (quickly approaching the E_Na_ value potential), subsequently inhibition of Na^+^ channels and activation of K^+^ ion channels (decreasing potential to values even below the original resting potential, after-hyperpolarization) and followed by the stabilization of the potential (back to the values similar to the resting potential).

For generation of action potentials, two processes influence the Na^+^ ion channels, first activation and deactivation of these ionic channels and then also inhibition and de-inhibition. The channels are able to pass the ions while they are activated and de-inhibited. At the beginning, the Na^+^ ion channels are in deactivated and de-inhibited state. The depolarization of the membrane potential increases the probability of Na^+^ channels activation. As one channel is activated, the influx of Na^+^ ions increases the potential more and other channels become also activated. However, the fast depolarization also leads to inhibition of Na^+^ ion channels, which thus no longer conduct Na^+^ ions. In addition, the K^+^ ion channels also become activated and allow the positive charge to exit the cell and the membrane potential to repolarize. The persistence of the K^+^ current for a few milliseconds following the action potential generates the after-hyperpolarization, during which inhibition of the Na^+^ channels is removed^16^. The temporal overlap between Na^+^ and K^+^ currents during an action potential was found to be significantly reduced to make the whole process more energy efficient^48^.

In most cells, each action potential is started somewhere between the region of the neuronal cell body where the axon originates, the axon hillock, and the axon initial segment^49–51^. The cytoplasm in the vicinity of the axon hillock is deficient of Nissl substance (a combination of stacks of rough endoplasmic reticulum interposed with rosettes of free polysomes) generally present at the soma but with unknown function^16^. The axon initial segment is the region adjacent to the axon hillock. Herein, microtubules generally form characteristic parallel fascicles not seen elsewhere. The axon initial segment is a small compartment with the lowest threshold for action potential generation due to high density of (voltage-sensitive) Na^+^ channels. Once a spike is initiated (e.g., about 30–50 µm from the axon in cortical pyramidal cells), this action potential is then propagated in both directions – down the axon, causing the release of neurotransmitter in the synaptic terminals, as well as back through the cell body, potentially modulating intracellular processes in the cell’s dendrites^50^. The axon initial segment and, to some extent, the axon hillock also have a specialized thick electrondense coating with a complex ultrastructure and unknown function^16^ separated by 5–10 nm from the inner surface of the membrane.

### Inhibitory Post-Synaptic Potentials (IPSPs)

Although an increase in K^+^ mediates IPSPs at some inhibitory synapses, in others, like inhibitory interneurons for the spinal motor neuron^52^, the IPSPs are due to a selective increase in Cl^−^ conductance. When the receptors on postsynaptic membrane bind the neurotransmitter, here the glycine (although the most common transmitter associated with inhibitory actions in many areas of the brain is GABA), they become selectively permeable to Cl^−^. For example, in spinal motor neurons, the membrane potential moves from a resting value of −65 mV toward the Cl^−^ equilibrium potential of approximately −70 mV as a result of the increase in Cl^−^ conductance.

### Active Spread of Signal in Myelinated Axon, Refractory Period for Unidirectional Spread of The Signal

Passive spread of the signal in the axon would allow effective spreading of the potential for up to several millimeters, which is not enough in comparison to the known length of axons (the largest ones having the length in meters in vertebrates like human or giraffe^17^). To enhance the conductance properties, it is necessary to either increase their diameter (with up to millimeter-scale values like some axons in invertebrates), which would be not suitable for mammalian brains, or increase membrane resistance (like in vertebrates). However, in the latter case, to keep the passive spreading of the signal fast, this has to be followed by decrease in axon membrane capacitance. The way the nervous system does this is through special satellite glial cells – oligodendrocites (in central nervous system) or Schwann cells (in peripheral nervous system). These cells wrap many layers of their plasma membranes, called together myelin sheaths, around axonal segments and maintain them^53,54^. The evolution of the insulating myelin sheath then allows for 10- to 100-fold increases in conduction velocity through axons of fairly small diameters. The bare portion of the axon at the end of each myelin segment, the node of Ranvier, has a complex structure. The density of voltage-sensitive Na^+^ channels at the node is high (10,000 µm^−2^) in comparison to that in the internodal membrane (20 µm^−2^). This difference in density means that the impulse is only actively generated at the node. A myelinated axon therefore resembles a passive cable with active booster stations – the impulse jumps from node to node, hence the process is called *saltatory conduction*. This allows axons of small diameters to conduct extremely rapidly^55^ and may extend over considerable lengths; for example, in a 20 µm-diameter axon conducting at 120 m·s^−1^ an impulse of 1-ms duration is transmitted over a 120 mm length of axon, including more than 100 nodes of Ranvier. The impulse is probably generated simultaneously by many nodes spreading their summed currents to the next adjacent nodes to activate them^16^.

Nevertheless, for very small and short axons, i.e. less than 1 µm in diameter, it is more effective to be unmyelinated^56,57^. The axon is generally unmyelinated in local circuit neurons (such as inhibitory interneurons). In unmyelinated axons, the Na^+^ and K^+^ channels taking part in action potential generation are distributed uniformly along the axon.

Due to the inhibition of Na^+^ channels, an action potential cannot be generated immediately after the generation of the previous one. The period of inhibition of Na^+^ channels is also accompanied and followed by after-hyperpolarization from the opened K^+^ ion channels, requiring another increased injection of ionic current into the cell to exceed the activation threshold again for any subsequent action potential. The practical implication of these refractory periods is that action potential from axon initial segment and subsequently from nodes of Ranvier spreads unidirectionaly – the nodes down in the direction cannot reactivate the nodes back on the path during the spread of one action potential.

## Chaotic oscillators

If the neuronal cell is able to integrate the input potential in the soma, then the changes of the resistance of the input paths can affect the increase or decrease in the frequency of generated action potentials. All this processing is also preceded by dendritic signal preprocessing operations. However, upon presenting a completely new, unknown input, the unit should be able to output a highly different response, but the documented dendro-dendritic range of operations is insufficient for this purpose as it is limited to adjusting the properties of the incoming signal only. To clearly distinguish the new pattern of input signal, the preferred response would be, for example, to exhibit a highly dissimilar signal on the output. The concept of high sensitivity to the input signal, but still of deterministic nature, is known to be employed in chaos theory. A nonlinear dynamic system is called chaotic if it is deterministic and highly sensitive to initial conditions^58^. The output of such a system for given initial conditions can exhibit a response similar to stochastic processes. The set of values the system tends to evolve, the system attractor, represents the responses the system is able to provide. Units capable of highly complex information processing should be able to exhibit chaotic behavior in relation to selected signal quantity, like amplitude, frequency or fire pattern (the time intervals between subsequent pulses) and thus to provide a wide set of different responses in relation to changes in such a selected quantity. The basic concepts to prove chaoticity^59^ in discrete dynamic systems include the numeric methods of determination of Lyapunov exponents (e.g. ref. ^60^) or the determination of correlation dimension (e.g. ref. ^61,62^). If the dominant Lyapunov exponent is positive, then the system presents highly divergent responses in time for very close inner states. Similarly, if the correlation dimension of attractor is determined to be greater than zero, then the system is able to present an uncountable set of responses, therefore the system is chaotic at least.

Oscillators exhibiting chaotic behavior in some selected quantity of signal, like its amplitude or frequency or fire pattern, are called chaotic oscillators. If the processing unit is behaving like an oscillator, and the processed information is coded by changes in given selected quantity of signal, then sufficient spectrum of oscillator’s responses is required, for example, chaotic behavior of such units, at least for some values of the processed quantity of the signal. Similarly, for aggregation of such units, to grow the number of responses of such a system exponentially or more with the number of aggregated units, for each base unit being a chaotic oscillator is also the necessary condition.

To construct the chaotic oscillator the theoretical concepts are well known – even three variables, one providing positive feedback, one providing slower positive feedback and one providing negative feedback, could form a simple chaotic oscillator^63,64^. And thus to prove the chaotic behavior of a given nonlinear dynamic system, in the oscillator assembled from simple circuits, at least two excitatory units and one inhibition unit must be included.

## Existing Circuit-Based Models of Neuron, Chaotic Oscillators Used as Neuronal Models

Many biological models have been inspired by circuit theory, especially after the important works of Rall^34,35,65,66^, as they try to combine the relative simplicity of electrical circuit simulation with observed spreading of signal in nerve cells. The whole-cell biological multi-compartment models provide detailed description of changes in concentrations of ions and potentials at different places of neuron^67–69^ and allow biologically relevant simulations at the cost of high computing complexity. Each biological structure of the cell is there often represented as a complex circuit of several components. One way to reduce the complexity of the model is to minimize the number of used electrical parts for each structure trying to preserve the biological relevance. Other way is to reduce the number of simulated compartments to only a few or to only one “generalized” compartment in single-compartment models. The complex and biologically plausible single-compartment models^12,70^ are often reduced by modification or simplification of underlying differential equations, where such models become also phenological only (^8,10,71^, etc.) – describing the outputs very similarly to the real neurons but without any biological justification or any proof-of-concept in circuit theory.

The simple single-compartment neuronal models constructed de novo with only small number of biologically plausible parts are based on a capacitor, switches or their more complex equivalents (Schmitt trigger, etc.), voltage source and resistors (the integrate-and-fire model, Lapicque 1907^72,73^, see ref. ^2,74^ for review). To provide an extended electric geometry of the fire pattern signal in such model, more complex parts are then also included (van der Pol-Bonhoeffer–FitzHugh–Nagumo model^75–78^), like inductors or sets of capacitors, without any subsequent biological explanation for such usage. Relative insensitiveness of the brain to magnetic fields (e.g. MRI screening methods) can for example probably disqualify inductors and thus RLC circuits at all^77,79^ from any such biologically plausible models.

There are many known chaotic oscillators based on electric circuits^80–85^. For neuronal models based on the reduction or adaptation of such circuits, it is very ease to diverge from biologically plausible parts in the model to previously used complex electric parts required by such oscillators, like transistors, operational amplifiers, etc.^77,86^, and lack to add any biological justification or description of any analogous mechanism present in a neuronal cell.

To include electrotonic structure of the neuron and realistic signal geometry properties of the fire patterns, the minimal model based on biologically plausible parts used, like the integrate-and-fire model, was also thought to consist of at least two compartments (e.g., ref. ^87,88^). Such two-compartment models were able to capture essential aspects of spike-firing patterns, as well as a wide range of regular spiking responses, including repetitive bursting^9^. Up to our knowledge for biologically plausible few-compartment models, the description of model variant for inhibitory neuron to meet the minimal requirements for forming a simple chaotic oscillator has also never been reported.

In this paper, we present a single-compartment model of excitatory and inhibitory neurons, the capacitor-switch model (CS model) of excitatory and inhibitory neurons, capable of simulating common fire patterns like regular spiking or repetitive bursting behavior in relation to electrotonic changes in the potential, but also using only electrical parts with identified biological counterparts of similar assumed function in neuronal cells. Later we show that few such inter-connected units are able to form a chaotic oscillator and provide a highly complex range of responses to given input signal based on its fire pattern.

## Results and Discussion

### The proposed CS model of neuron

#### CS model of the excitatory neuron

**Figure 2:**
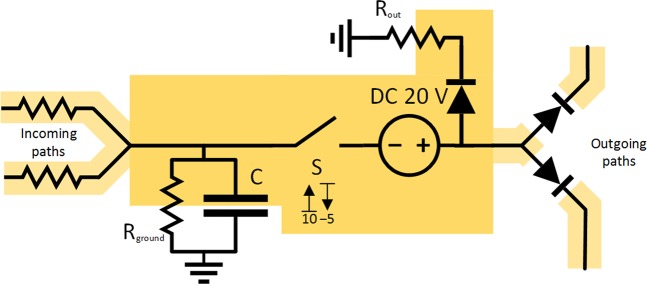
Final scheme of CS model of excitatory neuron consisting of capacitor C, resistor R_ground_, voltage-gated switch S, DC source, trashing path and outgoing paths to other units consisting of diode and resistor (drawn as part of incoming path). For the demonstration of principle only, the threshold values for voltage-gated switch S are selected as *V*_**S**, upper bound_ = +10 V, *V*_**S**, lower bound_ = −5 V, the DC_withdraw_ source voltage as *V*_DC withdraw_ = +20 V.

In Fig. 2 a scheme of electrical circuit for the CS model of excitatory neuron is presented as an extension of classical integrate-and-fire model. The circuit includes several simple electrical components – a capacitor, a voltage-gated switch, a floating DC source, a trashing path and outgoing paths to other units consisting of a diode and a resistor. The presented circuit integrates the incoming potential through the voltage of the capacitor and, in case of reaching the upper voltage bound, the connected DC source completely discharges the capacitor and any incoming current. The circuit allows any outgoing path to end even in the same unit and become recurrent.

To monitor the threshold at the capacitor, a special electrical part, a voltage-gated switch, was added. The voltage-gated switch in its blocking (open) state waits until the voltage on its input reaches the assigned upper voltage bound (*V*_S, upper bound_ parameter, the initial threshold) and then switches to its conducting state (closed). The switch remains closed until the voltage on its input (and similarly on the capacitor) reaches the assigned lower voltage bound (*V*_S, lower bound_ parameter, the after-hyperpolarization bound) and then it switches back to the open state. The upper bound value is defined above the ground potential (the resting state), and the lower bound potential is defined below the ground potential. The source voltage (*V*_DC withdraw_ − the difference between action potential and initial threshold) plus the assigned lower voltage bound are kept to be greater than the assigned upper voltage bound. Keeping this condition allows to completely discharge the capacitor to the assigned lower voltage bound not only through the trashing path but also through outgoing paths even if neighboring units have their capacitors charged to the voltages near the upper voltage bound. The used part differs for example from often used Schmitt trigger^76,89^, that it is only a passive part allowing to pass the current through with the same voltage as on its input.

From the perspective of circuit theory, the added R_ground_ path represents the currents delivered by ionic pumps reverting the potential back to the resting state and could also function as a simple shunt path (crowbar), but a very inefficient one, due to high resistance of R_ground_ resistor. This path is able to discharge the capacitor if there is no current on the incoming paths and high voltage on the capacitor (emulating failed initializations in neurons) or, in case of negative voltage on the capacitor, it allows recharging the capacitor up to zero level.

The ratio of R_out_ resistance to the resistors on the output paths and voltage on their targets determine the charge passed to each path. To support the R_out_ path and accelerate the decrease of the potential, there could also be another DC source on this path, however, this could be similarly emulated by significant decrease of R_out_ in comparison to resistors on other paths.

#### Membrane simulation and ionic pumps in our model

Many similar models of the cell membrane, including the integrate-and-fire model, use parallel connection of capacitor and resistor and we also keep this scheme – the capacitor as a membrane capable to separate two environments with different potentials and the ionic pumps trying to maintain the resting potential to some base level, a reference point. For such a reference point, the ground is used in circuit theory and thus one side of capacitor and resistor in our model is connected to the ground. This grounding is not related to the potential of extracellular space or the real ground. It only represents the potential which ion pumps are focused on, i.e. the resting potential. The active transport of ionic pumps is slower in order of magnitude than changes in potential caused by short openings of passive ion channels based on passive flow of ions down the concentration gradient. This disproportion could be reduced by comparatively larger numbers of ionic pumps relative to ion channels present in the cell. The process of reverting back the cell soma potential to the values of resting potential by ionic pumps is simulated through the added resistor in the model of a high resistance in comparison to any other resistors added later, which results in slow charging or discharging of the capacitor. As we focus only on the changes in overall potential in the model, we do not separate the ionic pumps by ions as is seen in many existing models. The energy of active transport required by ionic pumps is discussed after the next section.

#### Action potential in the model of excitatory neuron

**Figure 3:**
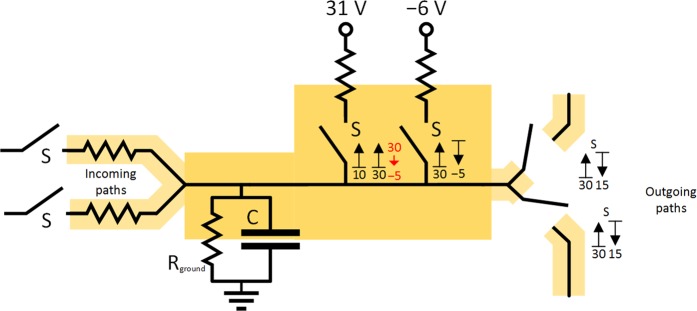
Draft modeled scheme of excitatory neuron with respect to increase and decrease in action potential caused by different ions depicted as two voltage-gated switches connecting two diferent sources. The condition required before reactivation of the same source is depicted in red. The synapses are represented as voltage-gated switches at the beginning of incoming paths and at the end of outgoing paths. The values of potential and voltage are for demonstration purposes only.

Fast depolarization followed by after-hyperpolarization in the potential are originated through the passive flow of the Na^+^ and subsequently K^+^ ions between two environments with high concentration gradient of these ions. Even though the transport is passive down the concentration gradient, the energy has been consumed as ATP for ATPase ionic pumps, which will have to pump the ions back to maintain the concetration gradient. The sharp increase in the potential originates in the exponential number of ionic channels activated. In electric circuits, the energy-fueled sharp increase and sharp decrease in potential is the function of a pulse DC source and we also added such a source to our model. The action potential is generated after reaching the threshold and for that case we can add two voltage-gated switches connecting different sources of voltage (see Fig. 3). Then the simulated action potential (*V*_action_ = 30 V) can be generated by connection of the first voltage source, DC_action_ (*V*_DC action_ = 30 V). To prevent immediate voltage increase up to the action potential, slightly higher voltage (e.g. 31 V) behind a resistor could be used instead. Once the V_action_ value is reached, the source is disconnected and the second source, DC_hyperpolarization_ (*V*_DC hyperpolarization_ = −5 V or −6 V behind the resistor), is connected. After reaching some *V*_hyperpolarization_ (here *V*_hyperpolarization_ = −5 V) on the capacitor, this source is also disconnected. After the end of action potential, the capacitor is left with the potential of *V*_hyperpolarization_, which is subsequently reverted back to the resting potential through the ground behind the R_ground_ resistor.

Let us think of the situation again. Once the DC_action_ source is connected, the charge on the capacitor C with capacitance *C*_C_ and voltage *V*_threshold_ will increase immediately or after a short time by Δ*Q* = *C*_C_ · (*V*_action_ − *V*_threshold_). When the V_action_ is reached, subsequent switches to output paths are opened and DC_hyperpolarization_ is also connected. All the charge of *Q* = *C*_C_ · (V_action potential_ − V_hyperpolarization_) is removed from the capacitor and distributed through the path with the DC_hyperpolarization_ source and other opened output paths. The voltage on the capacitor falls from V_action_ down to V_hyperpolarization_ and once the V_hyperpolarization_ is reached, the DC_hyperpolarization_ is disconnected and all other output paths are also closed. The charge from the capacitor is distributed according to voltages to the targets and resistors on their paths of all simultaneously opened paths from the capacitor. The similar situation could be established in another way, instead of two different connected sources, one voltage-gated switch and one floating source DC_withdraw_ (*V*_DC withdraw_ = *V*_action_ − *V*_threshold_ = 20 V) could be used together with one trashing path connected through the R_out_ resistor as in Fig. 2. When the potential of *V*_threshold_ is reached on the capacitor, then the switch connects the voltage source and *V*_threshold_ + *V*_DC withdraw_ is presented on its output. The charge drawn from the capacitor is *C*_C_ · (*V*_threshold_ − *V*_hyperpolarization_), which is less than in the previous case, however, this charge is also distributed according to resistances and potentials of all simultaneously opened output paths, including the trashing path. The correct setting of R_out_ and potential at the voltage source of the trashing path (e.g. 0 V for the ground) then allows distributing the same charge to all other output paths the same way as in the previous case. For the trashing path, the distributed charge will then be exactly Δ*Q* = *C*_C_ · (*V*_action_ − *V*_threshold_) lower than in the previous case. If any simulation is not dependent on the original values of distributed charges for the trashing path, then we can use this substitution without loss of generality. Such substitution will also have another effect. In the previous case, at the beginning of the action potential, when the DC_action_ is connected, any input signal at the input paths trying to charge the capacitor should have at least V_action_ not to be blocked by the action potential itself. If we employed the two-compartment model with some capacitor at the dendrite compartment, then the input signal could charge the capacitor in the dendrite compartment and, after the action potential, in the soma compartment, this charge could also be later transferred to the soma. Here, with the one floating DC_withdraw_ source, the voltage on the capacitor is V_threshold_ or less all the time and thus input signal from any other unit generating the action potential could enter the capacitor even when the current unit is already generating the action potential. And thus, the synchronized action potential of several units on one long path will not block either of them.

#### Synapses, dendrites, axon and further reduction of complexity in the model

For simulation of synapses, the voltage-gated switches on the incoming paths (or on the outgoing paths) can be added, emulating the passing of the charge only in case of spikes delivered from preceding units (or to succeeding units, respectively) (see Fig. 3). Assuming that the lower and upper voltage bounds for the inner voltage-gated switch and the synaptic voltage-gated switches on the output paths are set very similarly, differing only in addition of voltage of DC source (*V*_DC withdraw_), then the function of voltage-gated switches on the output paths of the same unit is reduced only to a) maintaining the direction of discharge from the capacitor of the unit to capacitors of neighboring units down the outgoing paths and b) not allowing any mutual bridging among the outgoing paths. For these two cases, we can further reduce the complexity of the model by replacing the synaptic input voltage-gated switches with ordinary diode elements on the output paths, leaving only the inner voltage-gated switch before the DC_withdraw_ source, as depicted in Fig. 2).

The axon allows the active spread of the signal using nodes of Ranvier. However, since these nodes only repeat the signal actively and unidirectionally to pass it through the resistors on one path, which can be simulated by additional inner voltage-gated switches, floating DC sources and diodes, we reduce this representation to use only one floating DC source for one unit like in Fig. 2.

Even though the dendritic operation can be very complex, our constructed model assume that these operations can also be simulated later in the soma, or that these operations could be at least simulated by aggregating more modeled units in sequence. Thus the path from synapse to soma is modeled here as a simple cable path with one resistor only. If some amplification of the signal is also required, a small floating-voltage DC source could be added.

#### CS model of the inhibitory neuron

**Figure 4:**
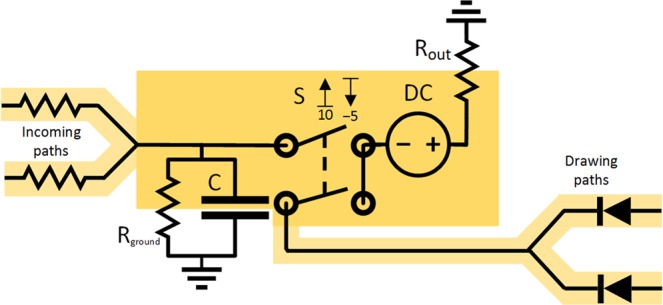
Final scheme of CS model of inhibitory neuron consisting of capacitor, DPST voltage-gated switch S, DC source, trashing path and drawing paths. Drawing paths to other units consist of diode and resistor (drawn as part of incoming path). For the demonstration of principle only, the threshold values for voltage-gated switch S are selected as *V*_S, upper bound_ = +10 V, *V*_S, lower bound_ = −5 V, the DC_withdraw_ source voltage as *V*_DC withdraw_ = +20 V.

If the inhibitory unit somehow mirrors any given excitatory unit using only very simple parts, then there are few theoretically apropriate schemes for such circuits. To provide negative voltage on the output, we designed the circuit presented in Fig. 4, a slight modification of the circuit in Fig. 2. This circuit uses additional voltage-gated switches (on its drawing paths) that are controlled by the potential of the capacitor of the unit, however, when they are closed, they are able to draw the voltage from any other connected capacitor, i.e. from any other unit. If we replace all the switches in the unit by only one main voltage-gated (multi)-switch (double pole single throw – DPST), then the mode of operation for the main voltage-gated switch and the subsequent switches will be the same. The DPST switch is also able to close several paths at once – the one connecting the capacitor of the unit with the DC_withdraw_ source and also the drawing paths with the DC_withdraw_ source – as depicted in Fig. 4.

The drawing path can be connected as any other incoming path to other units to inhibit the potential on their capacitors provided that the activating conditions are modified for the switches on these paths in the same way as the existing activating conditions for the main DPST switch, or that they are generally replaced by diodes on these paths. The original output node is grounded by the trashing path to trash the drawn potentials, however, if appropriate, there could also be regular non-trashed output paths. This inhibiting circuit is able to increase its activity with increasing activity on the incoming paths and is independent of the activity on the drawing paths.

#### Action potential in the model of inhibitory neuron

**Figure 5:**
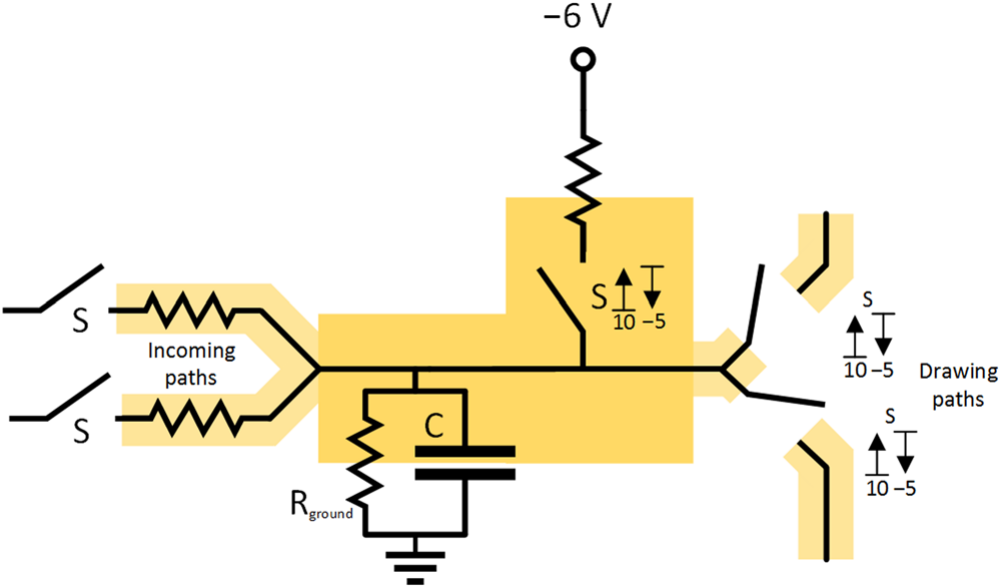
Draft modeled scheme of inhibitory neuron with decrease in potential caused by specific ion channels. The values of potential are for demonstration purposes only. The synapses are represented as voltage-gated switches at the beginning of incoming paths and at the end of outgoing paths.

Figure 5 depicts the electrical scheme of inhibitory neuron provided that an inhibiton potential originates in the decrease of potential to defined *V*_hyperpolarization_ value (*V*_hyperpolarization_ = −5 V in the model). Once the *V*_threshold_ potential is reached at the capacitor, the DC_hyperpolarization_ voltage source (−5 V or −6 V behind the resistor) is connected, removing all the charge from the capacitor and some other from the drawning paths. The activating condition for synapses on drawing paths (inhibitory) and regular output paths (excitatory) generally differs in the draft schemes presented, which is in correspondence to existing differences between inhibitory and excitatory synapses in real neurons. In the draft scheme, we can also make a substitution similar as for the excitatory neuron. A scheme depicted in Fig. 4 is able to simulate the same voltages on the capacitor and the same currents going through the drawning paths. Once the new floating source DC_withdraw_ (*V*_DC withdraw_ = 20 V) is connected, all the charge is removed from the capacitor, leaving it with *V*_hyperpolarization_ potential as before. To be able to reach this value, the *V*_DC withdraw_ has to be at least −1 · *V*_hyperpolarization_ (or more for any resistors behind) to discharge the capacitor down to the *V*_hyperpolarization_ potential. The resistances on the drawning paths must be then adjusted to simulate the same currents leaving through drawning paths as in the previous scheme, and the only difference in simulated current is left for the path containing the R_out_ resistor (the trashing path).

### An electrical circuit consisting of one or more CS models of neuron can produce spiking behavior similar to that recorded in real neurons

**Figure 6:**
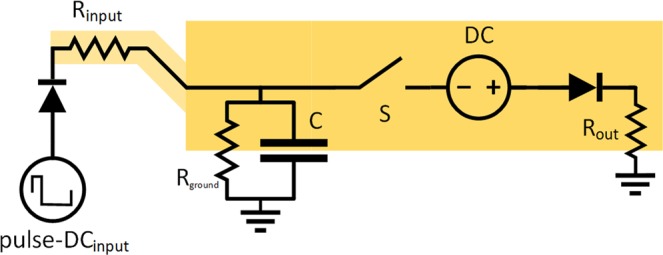
Scheme used for measuring output potentials – a scheme of excitatory neuron with the preceding pulse DC source to emulate a simple source of the signal.

**Figure 7:**
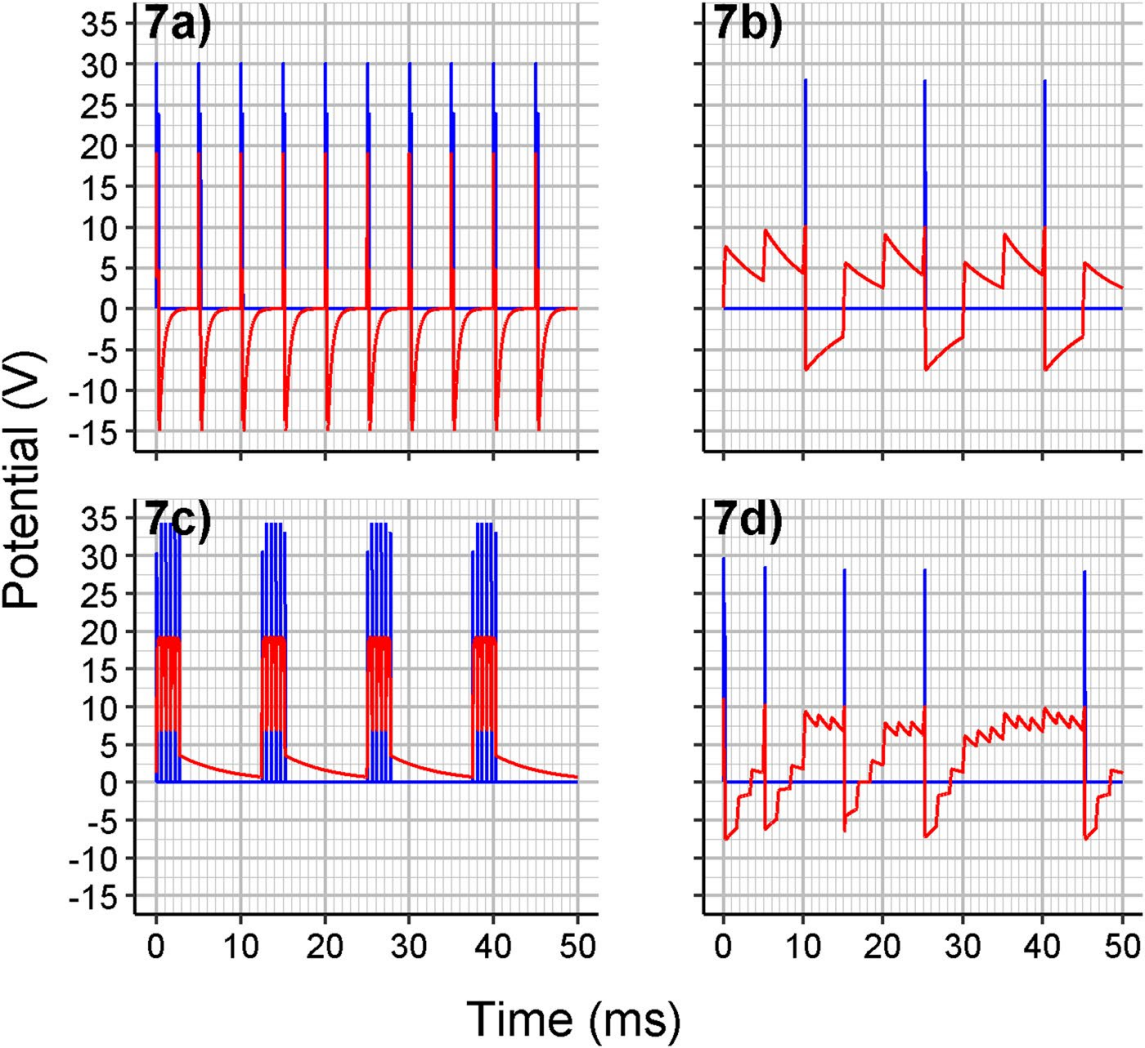
(**a**) Voltage on capacitor C (red) and potential on the entry point of resistor R_out_ (blue) for using the parameters set as in Table 1 (Value Set 1). For slightly higher capacities, the recharging even does not reach the rest state before the next spike. (**b**) For very high capacities of C or high resistances of R_input_, several pulses are required to reach the *V*_S, upper bound_ threshold voltage. The potential depicted is for R_input_ resistance of 80 Ω and other values set as before (with peaks much higher than upper voltage bound for the switch being the artifact of the simulation). (**c**) Bursting pattern behavior observed for the capacitance of C = 6 µF, DC_input_ frequency = 80 Hz, DC_input_ duty cycle = 20%, threshold voltage *V*_S, lower bound_ = 8 V, switch S refractory period *T*_S, min off delay_ = 0.5 ms and other parameters set as in Table 1 (Value Set 1). (**d**) Spike frequency adaptation pattern produced at the unit receiving the input from three DC_input_ pulse sources (the full scheme is depicted in Supplementary Figure S1) and increasing the resistance of R_input,1_ (to one of the sources) from 10 Ω by 30 Ω after each produced spike. The resistances of R_input,2_, R_input,3_, to other sources, are set to 250 Ω. Each pulse source DC_input_ is also shifted in phase of emitted spikes for 120 degrees. The capacitance of C = 6 µF and all the other parameters for the unit and the sources are set as in Table 1 (Value Set 1).

To emulate a simple source of the signal, a pulse DC square-wave voltage source with a diode can be connected to the input of the CS model of excitatory neuron (see scheme in Fig. 6). The resulting potential (against ground) measured on the entry point of the resistor R_output_, along with the voltage on capacitor C, is then presented in Fig. 7a**)** using the parameters summarized in Table 1. Applying power laws to given electrical circuits, the resulting voltage on the capacitor and the potential on the R_out_ resistor could also be recorded using slightly different parameter sets, or at least the same fire patterns (the time intervals between subsequent pulses) could be obtained (see Table 1). The value of RC time constant for resistance of R_ground_ and capacitance of C and the properties of DC_input_ source affect the ability of the capacitor to charge to its base (zero-level) potential before the next pulse from DC_input_ source occurs. If the capacity of C is too high or the resistance of R_input_ is too high, then several pulses are required for reaching the upper bound (see Fig. 7b**)**). In such a case, too small values of R_ground_ resistance would discharge the capacitor to zero level potential before the next pulse occurs and thus never reach the upper bound, which is observed as silent fire pattern.

**Table 1:** Parameter sets used for simulation of potentials depicted in Fig. 7a): the one used (Value Set 1), the alternative one (Value Set 2) to produce the same potentials, and the one (Value Set 3) to produce the same fire patterns as in Fig. 7a).

Parameter		Parameter sets and their alternatives for the same voltage on C and potential on R_output_ as in Fig. 7a)		Similar parameter set for at least the same fire patterns as in Fig. 7a)*
		Value Set 1	Value Set 2	Value Set 3
DC_input_ max voltage		+20 V	+20 V	+10 V
DC_input_ frequency		200 Hz	200 Hz	200 Hz
DC_input_ duty cycle		5%	5%	5%
R_input_ resistance		6 Ω	60 Ω	6 Ω
R_out_ resistance		10 Ω	100 Ω	10 Ω
The base unit	R_ground_ resistance	1000 Ω	10 000 Ω	1000 Ω
	C capacitance	500 nF	50 nF	500 nF
	S upper bound threshold voltage (*V*_S, upper bound_)	+10 V	+10 V	+5 V
	S lower bound threshold voltage (*V*_S, lower bound)_	−5 V	−5 V	−2.5 V
	DC_withdraw_ voltage (*V*_DC withdraw_)	+20 V	+20 V	+10 V

The parameter sets are derived for diodes modeled by the ideal diode model. These would slightly differ for other models, like the Shockley diode model.

*The recorded potential could differ from the values in Fig. 7a) by a multiplication constant.

The bursting spike behavior (see Fig. 7c**)** could be easily produced temporarily by increasing the lower bound threshold voltage for the switch and including any non-zero refractory period for the switch. If the threshold voltage *V*_S, lower bound_ is increased (here from −5 V to +8 V) the voltage on the capacitor will remain high after the spike – until the next spike or until discharging by the R_ground_ path. If we add another parameter for the voltage-gated switch, the refractory period *T*_S, min off delay_ preventing any immediate reactivation of the switch, then the temporarily high current on the input path can result in several spikes in a train on the output path, as the switch is drawing the potential almost immediately and is then blocked for some time, until the next spike can occur. While Ca^2+^ ions are known to play a significant role in burst spiking, our model suggests that changing the sensitivity of K^+^ ion channels or their partial deactivation or inhibition could be one of the molecular mechanisms employed. In the CS model, if the threshold voltage *V*_S, lower bound_ or the refractory period *T*_S, min off delay_ has to be regulated, then real setting of any of these parameters would be highly dependent on the hardware implementation of voltage-gated switch and would be also probably controlled by some other physical quantity like the specific voltage on another input to the used part.

If we want to see some spike frequency adaptation pattern, a more complex scheme is required – an excitatory unit receiving input from three other units or three pulse DC sources, each on its own path (see Supplementary Fig. S1 for the scheme). The resistance of resistor on the selected path (R_input,1_), with an initial value of 10 Ω, is increased by 30 Ω after the end of each produced spike, while the resistances on the other paths (R_input,2,_ R_input,3_) are kept at 250 Ω. The fire pattern recorded at the unit is depicted in Fig. 7d**)**, exhibiting spike-frequency adaptation through reducing the value of input current to the capacitor.

For chaotic fire pattern, one modeled excitatory unit with only a few independent sources of regular periodic input signal is not able to produce such fire pattern. Either a chaotic fire pattern signal should be present on the input for such behavior or, for the regular periodic input signal, a positive and a negative feedback have to be at least provided.

### Several units of the CS model of neuron can form a chaotic oscillator and the relation of fire patterns in their input signals is an important factor for chaotic behavior to occur

**Figure 8:**
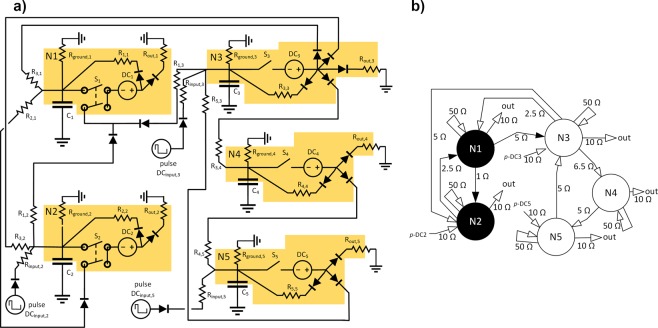
(**a**) Electric circuit of five interconnected units used to demonstrate chaotic oscillations. Two inhibitory units (N1, N2) are interconnected with three excitatory units (N3, N4, N5). DC_input,*i*_ pulse sources are connected to units N2, N3 and N5 through R_input,*i*_ resistors. (**b**) Schematic representation of the electric circuit with inputs, outputs, excitatory units and connections (white), inhibitory units and connections (black), and inter-unit path resistances.

To measure the response to input fire pattern, a scheme of three excitatory base units and two inhibitory units based on real neuron couplings observation^90^ was modeled as an electrical circuit (see Fig. 8). A central excitatory unit (N3 in Fig. 8) is cyclically interconnected with a chain of two excitatory units to provide a positive feedback to the signal at the unit and then with two other interconnected inhibitory units to provide the negative feedback. Three independent DC_input,*i*_ pulse sources are also provided to control the feedback characteristics. For this circuit, an output fire pattern from the interval of 0–3000 s was recorded using a discrete simulation with a fixed time step of 5 µs. An initial inconsistent fire pattern occurred only in a very short interval (approx. 10 ms) and thus was neglected. For each DC_input,*i*_ pulse source the max voltage was set to 15 V, DC_input,*i*_ frequency to 200 Hz if not stated otherwise, and DC_input,*i*_ duty cycle to 5%. For each excitatory and inhibitory unit the C_*i*_ capacitance was set to 6 µF, threshold voltage *V*_S, upper bound_ to +10 V, *V*_S, lower bound_ to −5 V. All R_ground_ resistances were set to 1000 Ω. All R_input_ and R_out_ resistances were set to 10 Ω. The resistances for R_*i*,*j*_ paths are depicted in Fig. 8b). The fire pattern was then measured on unit N3 all the time.

For the capacitance, DC_input_ pulse sources maximum voltage and frequencies used, one pulse is enough to charge the capacitor to reach the threshold voltage *V*_S, upper bound_, and thus the maximum detected interspike interval can be at most 5 ms or 1000 time steps of 5 µs (see also Table 2 and Table 3). The selected simulation time allowed us to measure at least 6 · 10^5^ points in the recorded fire pattern.

**Table 2:** Estimation of *D*_2_ correlation dimension using correlation sum based method and rotational spectrum based method for fire pattern recorded in electrical circuit as in Fig. 8 or with some components removed – Circuits with non-zero estimate for *D*_2_ dimension.

Circuit no.	DC_input,2_ frequency	DC_input,3_ frequency	DC_input,5_ frequency	Back synapses (R_i,i_ paths)	R_ground_ paths	Trashing paths (R_out_ paths)	N4, N5	Max. detected interval (in 5 µs time steps)	Estimated correlation dimension D2 including 90% confidence interval using correlation sum based method	Coefficient of determination R_2_ for regression used for estimating D2	Estimated correlation dimension D2 including 90% confidence interval using rotational spectrum based method	Coefficient of determination R_2_ for regression used for estimating D2
1	200	200	223	-	-	-	+	891	0.807±0.011	0.991	0.794±0.002	0.999
2	200	200	223	+	-	-	+	891	0.765±0.009	0.995	0.767±0.003	0.999
3	200	200	223	-	+	-	+	886	0.664±0.009	0.993	0.654±0.004	0.994
4	200	200	223	+	+	-	+	886	0.664±0.009	0.993	0.654±0.004	0.994
5	200	200	223	-	-	+	+	971	1.143±0.010	0.998	1.141±0.002	1.000
6	200	200	223	+	-	+	+	971	1.146±0.007	0.997	1.141±0.003	0.999
7	200	200	223	-	+	+	+	969	0.626±0.004	0.998	0.623±0.001	1.000
8	200	200	223	+	+	+	+	969	0.622±0.003	0.999	0.625±0.003	0.996
9	200	223	200	+	+	+	+	890	0.700±0.003	0.997	0.700±0.002	0.999

Back synapses ‘-’ value: All recurring paths with R_*i,i*_ and adjacent diodes are removed.

R_ground_ paths ‘-’ value: All R_ground_ paths are removed, the capacitor is left connected to ground.

Trashing paths ‘-’ value: All trashing paths (R_out_ paths) and adjacent diodes and grounding are removed.

N4, N5 ‘-’ value: Units N4 and N5 together with all their input and output paths are removed.

**Table 3:** Estimation of *D*_2_ correlation dimension using correlation sum based method and rotational spectrum based method for fire pattern recorded in electrical circuit as in Fig. 8 or with some components removed – Circuits with zero estimate for *D*_2_ dimension.

Circuit no.	DC_input,2_ frequency	DC_input,3_ frequency	DC_input,5_ frequency	Back synapses (R_i,i_ paths)	R_ground_ paths	Trashing paths (R_out_ paths)	N4, N5	Max. detected interval (in 5 µs time steps)
10	200	200	-	-	-	-	-	980
11	200	200	-	+	-	-	-	980
12	200	200	-	-	+	-	-	976
13	200	200	-	+	+	-	-	976
14	200	200	-	-	-	+	-	979
15	200	223	-	-	-	+	-	884
16	200	200	-	+	-	+	-	979
17	200	223	-	+	-	+	-	884
18	200	200	-	–	+	+	-	975
19	200	200	-	+	+	+	-	975
20	200	200	200	+	+	-	+	979
21	200	223	223	-	-	+	+	884
22	200	200	200	+	+	+	+	975
23	200	223	223	+	+	+	+	891

Back synapses ‘-’ value: All recurring paths with R_*i,i*_ and adjacent diodes are removed.

R_ground_ paths ‘-’ value: All R_ground_ paths are removed, the capacitor is left connected to ground.

Trashing paths ‘-’ value: All trashing paths (R_out_ paths) and adjacent diodes and grounding are removed.

N4, N5 ‘-’ value: Units N4 and N5 together with all their input and output paths are removed.

Attractor dimension in *R*^*N*^ is known to be equal to the attractor dimension reconstructed from one output in *R*^*W*^, where *W* ≥ 2 *N* + 1 is the length of the sliding window, i.e. the embedding dimension^91,92^.

Each simulated electrical circuit could be seen as a (discrete) dynamic system. The description of complete state of such a system would include the voltage in all nodes and current in all branches of the circuit. For description of complete state of circuit as depicted in Fig. 8, it is appropriate to remember the charge of each capacitor, the state of the switch in each unit and the global time (to determine the phase of cycle or voltage of each DC_input_ source). All the other parameters required for computation of complete description, such as resistance, DC_input_ frequencies or duty cycles are constant during the simulation. The dimension *N* is thus estimated as 11 for a circuit consisting of five units and 7 for circuits consisting of three units (see below), and the embedding dimension (sliding window) was then selected as *W* = 23 for all the circuits.

For the Lebesque measurable set *F* ⊂ *R*^*n*^, the correlation sum for given distance *r* > 0 is defined as^62^:

$$C(r)={\mathrm{lim}}_{N\to \infty }\frac{2}{N(N-1)}\mathop{\sum }\limits_{i=1}^{N-1}\mathop{\sum }\limits_{j=i+1}^{N}I(\Vert {x}_{i}-{x}_{j}\Vert \le r)=\mathop{{\rm{E}}}\limits_{{x}_{i},{x}_{j} \sim {\rm{U}}(F)}(I(\Vert {x}_{i}-{x}_{j}\Vert \le r))$$

where ||·|| denotes Euclidean norm, *I* is the indicator function, *E* is the mean value function, and *x*_1_,…, *x*_*n*_ are the vectors from *F*. The correlation dimension *D*_2_ of set *F* is then defined as^62,93^:

$${D}_{2}={\mathrm{lim}}_{r\to {0}^{+}}\frac{\mathrm{ln}\,C(r)}{\mathrm{ln}(r)}$$

Using another method^61^, rotational spectrum S(*Ω*) for given $\Omega \in {R}_{0}^{+}$ is defined as:

$$S(\Omega )=\mathop{{\rm{E}}}\limits_{{x}_{i},{x}_{j} \sim {\rm{U}}(F)}\left(\exp \left(-\frac{1}{2}{(\Omega \Vert {x}_{i}-{x}_{j}\Vert )}^{2}\right)\right)$$

and if correlation dimension *D*_2_ exists, then it can be computed as:

$${D}_{2}={\mathrm{lim}}_{\varOmega \to \infty }\frac{-\mathrm{ln}\,{\rm{S}}(\Omega )}{\mathrm{ln}(\Omega )}$$

**Figure 9:**
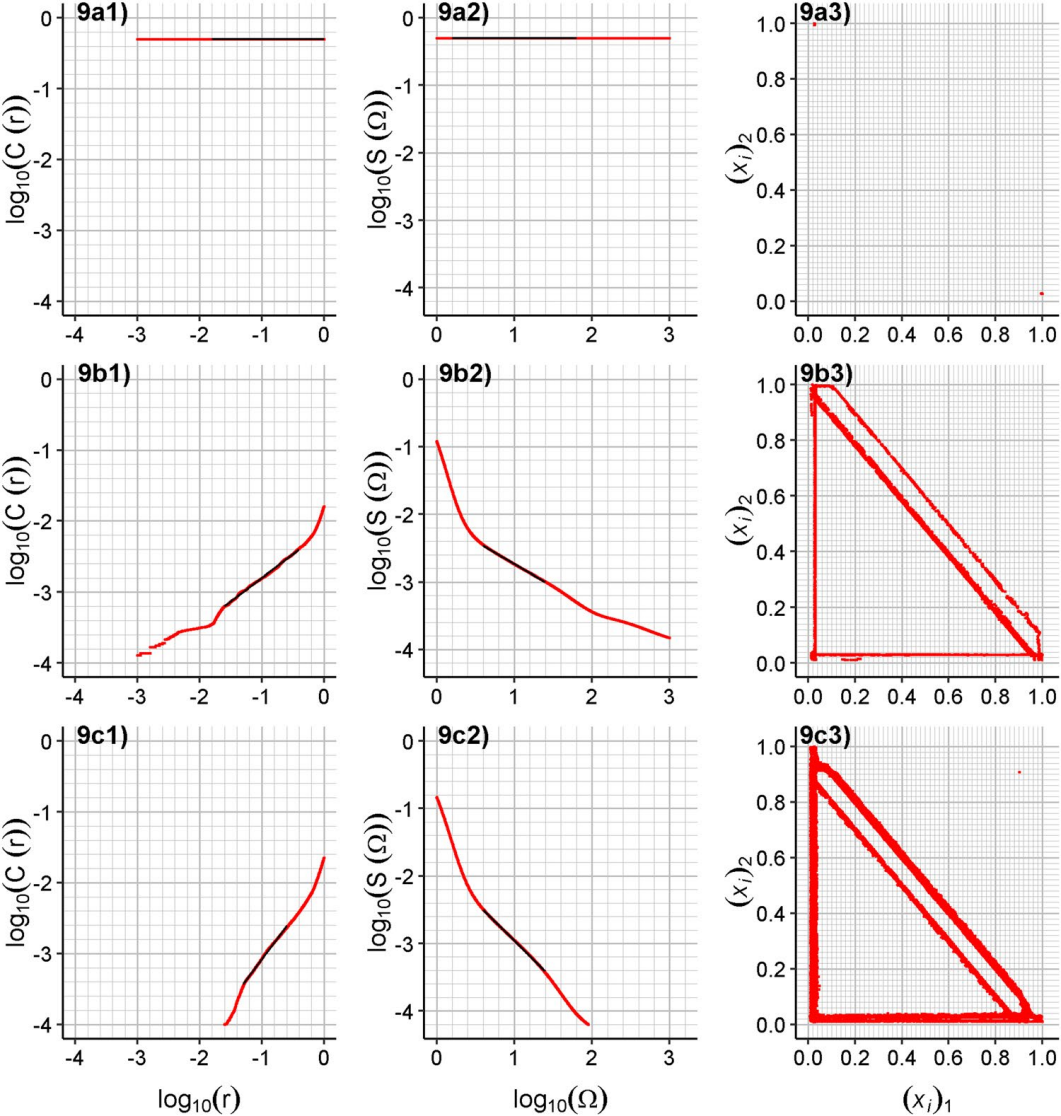
Determining the correlation dimension *D*_2_ for (**a**) circuit no.13, (**b**) no. 9 and (**c**) no. 6 using regression analysis. (1) log_10_(C(*r*)) vs. log_10_(*r*) plot (red) and fitted linear regression (black), (2) log_10_(S(Ω)) vs. log_10_(Ω) plot (red) and fitted linear regression (black), (3) Embedding of recorded values of fire patterns in two dimensions, where *x*_*i*_ are only two-dimensional vectors. For better readability of the plots, decimal logarithms were used instead of natural logarithms.

Using both methods the estimated *D*_2_ values differ a little. These differences are prevalently due to the discrete simulation of the system. The simulation also does not allow the presence of interspike intervals shorter than the time step. However, both methods are robust enough to prove the resulting *D*_2_ values being non-zero and thus confirm the chaoticity, or being zero for only a countable set of supposed responses. The selected cases for determination of *D*_2_ dimension, together with embedding in two dimensions, are depicted in Fig. 9.

For two circuits tested (no. 22 in Table 3, no. 8 in Table 2), the frequency of all pulse sources DC_input,*i*_ is set to 200 Hz, with the exception of DC_input,5_. For circuit no. 22 the DC_input,5_ frequency is also set to 200 Hz, for circuit no. 8 this frequency is set to 223 Hz. For a given circuit differing only in the frequency of selected DC_input_ source, the output fire pattern was able to demonstrate chaotic behavior for 223 Hz at DC_input,5_. To determine the other required components for reaching chaotic behavior, some parts were removed from circuit no. 8 and the *D*_2_ correlation dimension of fire pattern was estimated for circuits without back synapses (R_*i,i*_ paths), without *R*_ground_ paths, without excitatory units N4 and N5, without trashing paths (R_out_ paths), or with any combination of these restrictions. As can be seen from Table 2 and Table 3, even without R_ground_ paths, without back synapses, and without the trashing paths, the circuit is still able to exhibit chaotic behavior. However, if the part of the circuit consisting of N4 and N5 units (positive feedback based on the same or different frequency) is removed, the chaoticity is lost. Even if the input frequency of the measured unit (DC_input,3_) is different from the input frequency of negative feedback (DC_input,2_), the chaotic behavior is not present unless positive feedback on different input frequency is also present. The input signal frequency for measured unit (DC_input,3_) and positive feedback unit (DC_input,5_) is required to be different to keep the chaotic response. The input signal frequency for negative feedback unit (DC_input,2_) could then match any of these frequencies.

If the R_ground_ paths are present in an already chaotic oscillator, the *D*_2_ coefficients tend to be decreased, probably due to discharge of the small voltages of the capacitor to ground which results in slight smoothing of the fire pattern intervals. However, the selected *R*_ground,*i*_ resistances were not able to completely suppress the chaotic behavior at all. The effect of trashing paths seems to be a little pro-chaotic if they are present in an already chaotic oscillator, but not in all cases. The R_out_ paths were not able to evoke chaotic behavior in the absence of any other required component.

The *D*_2_ dimensions computed for circuits with and without back synapses are almost equal, which theoretically holds for connected ideal DC_*i*_ sources and thus it also confirms the numerical stability of the simulation. As another test of numerical stability, we can confirm that all the circuits with the chaotic output fire pattern keep this behavior and vice versa if we exchange the 200 Hz and 223 Hz frequencies, i.e. in circuits where all the original 200 Hz sources become 223 Hz sources and all the original 223 Hz sources become 200 Hz sources, or if we use the smaller time step of 1 µs. All the circuits also keep their chaotic or non-chaotic behavior for both diode model implementations used (the Shockley diode model with forward voltage at 1 A set to 805.9 mV, or the ideal diode model).

The proper coupling of excitatory and inhibitory units, with appropriate frequencies of input signal, is able to form a highly deterministic response (see also the projection of recorded fire patterns to two dimensions in Supplementary Fig. S3 and for selected cases in Fig. 9) presented in the form of their output signal fire pattern. The confirmed chaotic behavior then proves the high sensitivity of the presented system to the input signal – its frequency or, more generally, its fire pattern.

### Biological relevance of proposed CS model of neuron

Our electrical schemes use potentials and selected resistances to best explain the function of the circuit and, as can be seen from Table 1, these values could be simply scaled to better match the values measured on a biological neuron. The presented one-compartment model is able to show the oscillation patterns of regular spiking, bursting or spike adaptation. Similarly, if dendritic compartments maintain the potential independently of the soma, similar CS model units could also be located in dendrites or such units could be adapted a little to execute some other simple signal preprocessing operations. Even though such preprocessing operations are important, the fact that synapses at dendrites can be dynamically created or eliminated suggests that these operations improve the overall performance, but possibly are not fundamental to resulting information processing. This is assumed based on the fact that 1) diseases affecting axon conductivity, like demyelination at multiple sclerosis, have a much more detrimental effect on overall information processing and memory storage and 2) following high damage of brain tissue, many functions, for example for controlling the musculoskeletal system, can be reconstructed through adaptation of only a small fraction of undamaged tissue, generally not sharing the identical dendritic tree.

Nevertheless, using this function model, chemical synapses would represent a limiting factor for the frequency pattern of the output signal. The approximately 1 ms delay for reaching the potential to postsynaptic cell bottlenecks the processed frequencies of spikes in the signal to approximately 1000 Hz. To the best of our knowledge, even for pathologic states of the brain, such in the centers of epileptic lesions, no strong signals of any higher frequencies were recorded (e.g. ref. ^50^). Care should be also taken where signal processing methods like Fast Fourier Transform are applied to any non-harmonic recorded signal, like periodic pulse signal, reporting properly non-zero values for harmonic coefficients significantly higher than 1000 Hz^94^ (see also Supplementary Fig. S2) even if no such signal was originally present.

The electrical circuit consisting of many small conductive compartments separated by membrane clefts and synapses could also have several advantages over one always-connected system. In the compartmental system, the collapse of one cell or a compartment would not collapse the whole circuit – an alternative path in the highly interconnected system could be possibly found. Another advantage would be that the multicompartmental system is not dependent on exact values of capacitance in each of the compartments. The spread of potential, increased to the same or similar but controlled levels from presynaptic to postsynaptic terminal, allows to spread similar potential through many compartments differing in their capacitance highly – the ion channels simply raise the potential up to the required level at the expense of higher energy consumption in compartments with high capacitance. When seen as a robust cable path with booster stations to keep the spreaded potential, such a function is not in collision with our proposed model. Another function of the limited amount of neurotransmitter in the synapse or of meaningful resistances in the axon or dendrites could be to stop any high currents flowing through the circuit. This was also suggested as prevention of hyperexcitability, for example in cortical pyramidal neurons^95^. Last but not least, a role of intraneuron compartmentalization would also be the temporary preservation of the incoming signal received during the action potential. Indeed, the sharp increase in potential in only one whole cell compartment would block the input signal from any other cells. This problem could be overcome through integration of the signal in the preceding dendrite compartment. In our model, the preservation of the received input signal during the action potential is simulated by using a floating DC source instead of connecting the two DC sources of opposite potentials in fast sequence.

The simulation using electric circuits is based on spreading the changes in electropotential through metallic wires (3 · 10^8^ m/s), which is significantly faster than through solution of ions (in tens of meters per second, also reported as 120 or 150 m/s for saltatory conduction^16,22^). The speed of light allows these changes to propagate almost immediately, while the limited speed of saltatory conduction could cause some delay of signal. When comparing the speed of saltatory conduction with the theoretical limit recorded as approximately 1 ms delay of synapse switching, the resulting distance passed until the next possible fire (12 or 15 cm) is still enough to pass from one part of the brain to another. Even though the non-zero delay could affect other important features like synchronization of fire patterns, a simulation of similar systems showed^90^ that the signal delay is not as important as changes in path resistances for the resulting observed fire patterns. For this, a dynamic change of amount of neurotransmitter released to the synaptic cleft, or the protein machinery of Nissl substance with stacks of rough endoplasmic reticulum interposed with arrays of free polyribosomes, could play a supportive role.

## Conclusion

The constructed electrical circuit, the capacitor model of neuron, allows to generate common fire patterns occuring in neuronal cells including advanced fire patterns like repetitive bursting, previously known only from two-compartment models. In comparison to other models based on simple electrical circuits, only parts with equivalent biological structures of neuronal cells have been used and for this reason the usage of inductors, sets of capacitors or transistors has been avoided. As a necessity condition for formation of any simple chaotic oscillator responding to input signal frequency or fire pattern, both the excitatory and inhibitory units have been designed. The combination of these units has been then proven to work as a chaotic oscillator, and for a selected frequency of the input signal, it is able to generate highly different fire patterns in response. It has been later shown that the chaotic behavior of such an oscillator is dependent at least on the frequency of the input signals and the included inhibitory and excitatory units providing negative and specific positive feedback to the signal.

## Methods

### Software simulation of electrical circuits, statistical evaluation

All the circuit simulations were done using Paul Falstad’s Circuit Simulator 1.6i compiled in Java 11 for CentOS 7.6 and running on Cisco C220 M5 server computer (2 Intel Xeon Gold 6154 processors, 384 GB RAM). A time step of 5 µs was used (allowing approximately 1000 points for each DC_input_ source period) and the results were subsequently confirmed using the time step of 1 µs. All the data were subsequently evaluated using R software for Windows^96^.

### Determination of correlation dimension

The vectors in formulas *x*_*i*_, *x*_*j*_ are elements of [0.0, 1.0]^23^ and represent the embedding of the time series of determined interspike intervals in *R*^*W*^. This means (*x*_*i*_)_*k*_ is the *k*-th time interval in the *x*_*i*_ vector, where the detected interspike interval is normalized to [0.0, 1.0] by division by the maximum detected interspike interval of the series (see Table 2 and Table 3. For the maximum detected interspike intervals listed in Table 2 and Table 3 and for the time step of 5 µs, the normalized value is never below 10^−3^. Thus, to estimate the *D*_2_ correlation dimension of recorded fire patterns, the *r* values^62,93^ were selected linearly from the logarithm scale of [10^−3^, 10^0^], and similarly, the Ω values^61^ were selected linearly from the logarithm scale of interval [10^0^,10^3^]. For random 10^6^ pairs of points the C(*r*) and S(Ω) values were computed for each *r* and Ω value, and *D*_2_ was estimated together with the 90% confidence interval using the linear regression method for the central region of the curve, with the coefficient of determination *R*^2^ also computed (see Table 2). The values of *D*_2_ less than 0.03 were treated as zero. All non-zero values of *D*_2_ were also determined as significantly non-zero values (*p*-value at most 6 ∙ 10^−112^), confirming the chaoticity.

## Supplementary Information

Supplementary Information.
