# Supplementary material for: Simple capacitor-switch model of excitatory and inhibitory neuron with all parts biologically explained allows input fire pattern dependent chaotic oscillations
Source: Sci Rep. 2020 Apr 30;10:7353. doi: 10.1038/s41598-020-63834-7 (PMC7192907; doi:10.1038/s41598-020-63834-7)

SUPPLEMENTARY MATERIAL FOR THE PAPER:

Simple capacitor-switch model of excitatory and inhibitory neuron with all parts biologically explained allows input fire pattern dependent chaotic oscillations

Pavel Cejnar[1,*], Oldřich Vyšata[1,2], Jaromír Kukal[1], Martin Beránek[3], Martin Vališ[2], Aleš Procházka[1,4,*]

[1]Department of Computing and Control Engineering, Faculty of Chemical Engineering, University of Chemistry and Technology in Prague, Prague, Czech Republic

[2]Department of Neurology, Faculty of Medicine in Hradec Králové, Charles University, Hradec Králové, Czech Republic

[3]Independent researcher, Uppsala, Sweden

[4]Czech Institute of Informatics, Robotics and Cybernetics, Czech Technical University in Prague, Prague, Czech Republic

*** Correspondence:**
pavel.cejnar@vscht.cz, ales.prochazka@vscht.cz

Supplementary Material I: An excitatory unit exhibiting a spike frequency adaptation fire pattern

To the excitatory unit as in **Figure 2** were connected three pulse DC_{input} sources through the resistors $R_{input,1}$ = 10 Ω, $R_{input,2}$ = 250 Ω, $R_{input,3}$ = 250 Ω. The unit has C = 6 μF and all other parameters for the unit and the sources are set as in **Table 1** (Value Set 1). After the end of each spike produced, the $R_{input,1}$ resistance is raised by 30 Ω. The resulting fire pattern for the unit presented in **Figure 7d)** exhibits spike frequency adaptation. To emulate the incoming current flowing from more sources independently, each DC_{input} source is shifted in phase of emitted spikes for 120 degrees.

Figure S1: Electric circuit of unit receiving input signal from three other sources and exhibiting spike frequency adaptation. Three pulse DC sources form the input to the unit, each connected by its own path with resistor and shifted in phase by 120 degrees. The dynamic change of $R_{input,1}$ resistance results in spike frequency adaptation fire pattern for the unit.

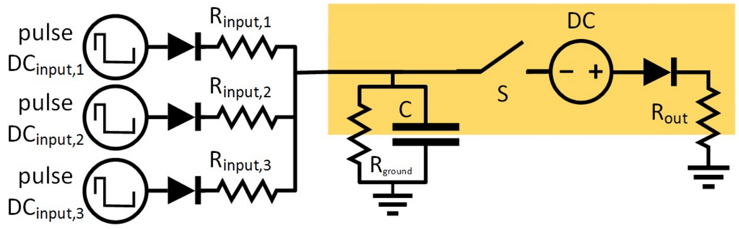

Supplementary Material II: Observed mix of output voltage patterns of a capacitor-modelled neuron shows a rich FFT spectrum similar to EEG

For the same configuration as in **Figure 6**, capacitance C = 6 μF and all the other parameters for the unit and the sources are set as in Table 1 (Value Set 1), the signal was recorded for an interval of 5.0 s with a time step of 5 μs and the Fast Fourier Transformation (FFT) was applied. For this signal with a DC_{input} source frequency of 200 Hz, the resulting harmonic amplitude coefficients for frequencies are depicted in **Figure S2a)**. Using the same configuration, four recordings were made for the same interval and time step but for different DC_{input} source frequencies (150 Hz, 180 Hz, 200 Hz, 223 Hz). The FFT was then applied to the sum of these four signals and the resulting amplitude coefficients for frequencies are depicted in **Figure S2b)**. For frequencies above approximately 2000 Hz the decrease of amplitude coefficients is almost linear in these graphs using log scale values. For square-wave signal like pulse or similar, the reconstruction of the signal using sinusoid-like signals leads to the presence of many high-frequency coefficients, even though no such signals were originally present. If the shape of neuron action potential is highly dissimilar to the sinusoid signal because of shape or the dynamic changes in frequency then the presence of high-frequency amplitude coefficients in FFT applied to electroencephalograms (EEG) does not mean that the sinusoid signal of given frequency was originally present.

Figure S2: FFT amplitude coefficients for frequencies for a) 5.0 s of a 200 Hz pulse signal and b) mix of 5.0 s of 150 Hz, 180 Hz, 200 Hz and 223 Hz pulse signals. The sampling interval and the time step allowed us to obtain coefficients for frequencies of up to 100,000 Hz with a 0.2 Hz step.

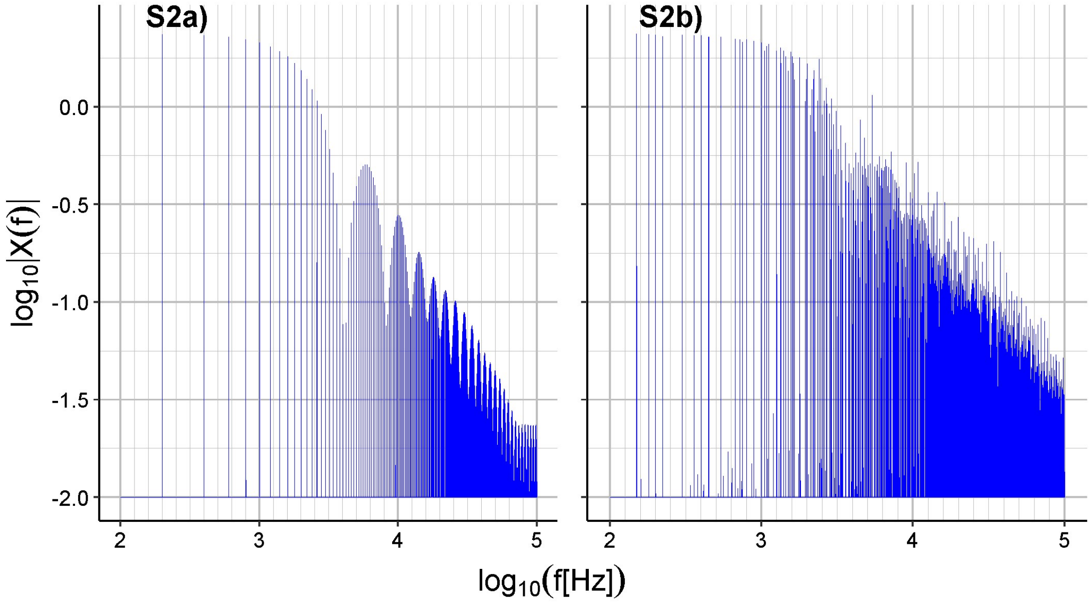

Supplementary Material III: Embedding of recorded values of fire patterns in two dimensions

Figure S3: **Embedded time series of determined interspike intervals (fire patterns) in R^2 for all units of all circuits from Table 2.**

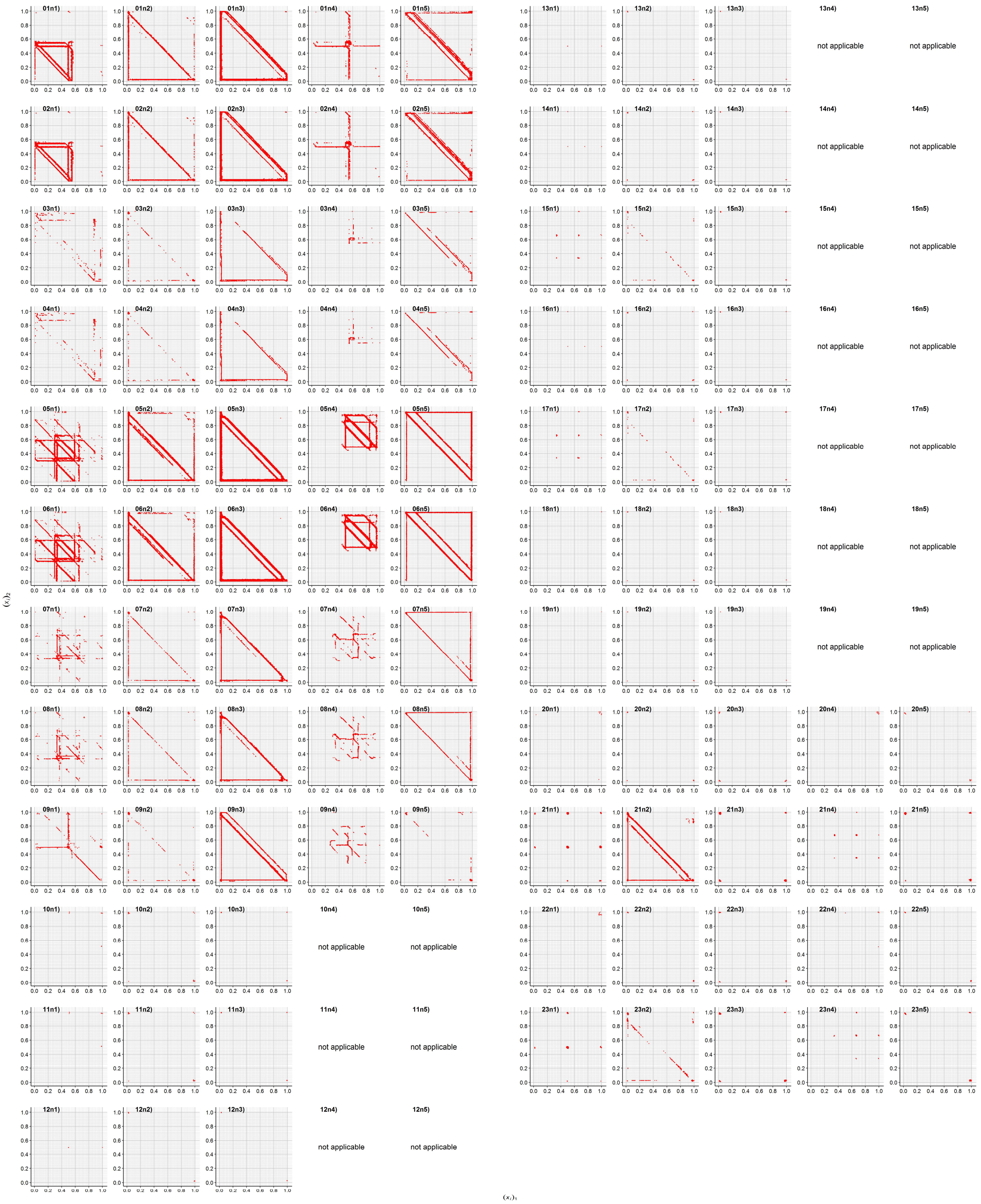

Supplement: Supplementary file 1 — Supplementary Information. [file 41598_2020_63834_MOESM1_ESM.pdf]
